# Are free anti-tuberculosis drugs enough? An empirical study from three cities in China

**DOI:** 10.1186/s40249-015-0080-y

**Published:** 2015-10-28

**Authors:** Shanquan Chen, Hui Zhang, Yao Pan, Qian Long, Li Xiang, Lan Yao, Henry Lucas

**Affiliations:** The Jockey Club School of Public Health and Primary Care, The Chinese University of Hong Kong, Hong Kong, China; National Center for TB Control and Prevention, China CDC, Beijing, PR China; The Third Affiliated Hospital, Sun Yat-sen University, Guangzhou, China; Duke Global Health Institute, Duke University, Durham, NC USA; Global Health Research Center, Duke Kunshan University, Kunshan, China; School of Medicine and Health Management, Huazhong University of Science and Technology, Wuhan, China; Institute of Development Studies, Sussex University, Brighton, UK; Chinese Center for Disease Control and Prevention, Beijing, PR China

**Keywords:** Tuberculosis, Economic burden, Catastrophic expenditure, Compliance, China

## Abstract

**Background:**

Tuberculosis (TB) patients in China still face a number of barriers in seeking diagnosis and treatment. There is evidence that the economic burden on TB patients and their households discourages treatment compliance.

**Methods:**

A cross-sectional study was conducted in three cities of China. Patients were selected using probability proportional to size (PPS) cluster sampling of rural townships or urban streets, followed by list sampling from a patient register. Data were collected using a questionnaire survey, key informant interviews and focus group discussions with TB patients to gain an understanding of the economic burden of TB and implications of this burden for treatment compliance.

**Results:**

A total of 797 TB patients were surveyed, of which 60 were interviewed in-depth following the survey. More than half had catastrophic health expenditure. TB patients with higher household incomes were less likely to report non-compliance (OR 0.355, 95 % CI 0.140–0.830) and patients who felt that the economic burden relating to TB treatment was high more likely to report non-compliance (OR 3.650, 95 % CI 1.278–12.346). Those who had high costs for transportation, lodging and food were also more likely to report non-compliance (OR 4.150, 95 % CI 1.804–21.999). The findings from the qualitative studies supported those from the survey.

**Conclusion:**

The economic burden associated with seeking diagnosis and treatment remains a barrier for TB patients in China. Reducing the cost of treatment and giving patients subsidies for transportation, lodging and food is likely to improve treatment compliance. Improving doctors’ salary system to cut off the revenue-oriented incentive, and expanding current insurance’s coverage can be helpful to reduce patients’ actual burden or anticipated burden. Future research on this issue is needed.

**Electronic supplementary material:**

The online version of this article (doi:10.1186/s40249-015-0080-y) contains supplementary material, which is available to authorized users.

## Multilingual abstracts

Please see Additional file 1 for translations of the abstract into the six official working languages of the United Nations.

## Background

Tuberculosis (TB) remains the most important infectious disease worldwide. Its etiological agent, Mycobacterium tuberculosis, infects one-third of the world population and it accounted for some 1.5 million deaths in 2013 according to WHO estimates [1]. China accounted for 11 % of global TB cases in 2013. It ranked fifth in terms of the epidemiologic burden of TB, with an incidence rate of 70 per 100,000, a prevalence rate of 94 per 100,000 and an estimated 3.0 deaths per 100,000 attributed to TB in 2013 [1]. Attaining WHO’s end TB strategy, zero deaths, disease, and suffering due to TB by 2035, will be a major challenge [2]. Non-compliance with treatment remains a central concern [3–6]. The direct observation of treatment (DOT) approach has proved effective, but, as in many countries, considerable proportion of non-compliant patients exist [6–12].

Studies indicated that education level, marital status, employment, and lack of TB and its treatment related knowledge were important reasons for non-compliance [8, 9, 13–15]. A number of studies also assessed related costs and found that a high economic burden is associated with poor treatment compliance [16–21].

To reduce the financial burden on and barriers to patients to facilitate seeking essential healthcare, a “free-TB service policy” has been implemented [9, 14, 16, 21, 22]. Under this policy, tuberculosis suspects are provided a free diagnosis and anti-tuberculosis treatment, including designated first-line TB drugs (6 months for new patients, 8 months if previously treated), chest X-ray examination for the first and last months, and sputum smear test [16, 21, 23–25]. Going with this policy, there are three service model: dispensary model, specialist model, integrated model [26]. The dispensary model is the most prevalent model. A TB dispensary is usually a department of Centre for Disease Control (CDC), with free treatment is only provided in this place. General hospitals are just responsible for referring TB suspects to dispensaries, and usually are supposed should not treat TB patients, unless severe or complicated cases. The specialist model is similar to dispensary model, with severe TB cases should only be treated in the specialist hospital, but treatment cost in specialist hospital is not covered by the free treatment policy. The integrated model is a new development, with the most popular general hospital in the district, usually called as TB designated hospital, provides TB treatment. TB dispensary continues to provide related service but just limited in training, mass education and case supervision, and should report and ensure referred TB suspects arrived in the designated hospital. Expenditure beyond the “free-TB service policy” was supposed to be covered by insurance. In china, health care system is bifurcated in nature between rural and urban areas [27], and TB patients mainly covered by government-led complementary insurance programs: the Urban Employee Basic Medical Insurance (UEBMI), designed exclusively for urban worker; the Urban Resident Basic Medical Insurance (URBMI), designed for urban residents without formal employment; the New Cooperative Medical Scheme (NCMS), designed for rural population.

Despite the free policy and the cover of insurance, the medical and non-medical costs incurred by TB patients and their households are still substantial [16, 28–32]. The phenomenon that requesting repeated investigation, including blood tests, X-rays and even CT test, and prescribing liver protection and ancillary drugs is not uncommon [10, 16, 21, 25, 33].

Several studies have also found that the anticipation of a high economic burden and concern with ‘sunk costs’ can affect patient decisions [9, 34–38]. Some patients may have poor compliance because they expect that the overall cost of treatment will be high even though their actual out-of-pocket payments have been relatively limited. Others who are facing serious financial difficulties may persevere with treatment because of the considerable investment in care already undertaken: the sunk cost effect. The observation of such complex relationships between economic burden and compliance emphasizes the need for a combination of qualitative and quantitative approaches. However, previous studies have generally adopted one approach. The primary objective of this paper is systematic research this problem through a mixed method.

Another gap is that previous studies usually focus on the direct medical cost. Few studies involve with direct non-medical and indirect costs. For certified low income and unemployed patients, just transportation cost can account for a considerable proportion of their annual household income. How about the relationship between economic burden incurred by direct non-medical and indirect costs and treatment compliance? The second objective of this paper is to fill this gap, and to examine whether and to what extent this burden affects patient treatment compliance.

## Methods

### Study setting

Since 2009, the Bill & Melinda Gates Foundation, one of which efforts in China is fighting HIV/AIDS and tuberculosis (TB), in collaboration with the National Health and Family Planning Commission of the People’s Republic of China (NHPFC), known as the Ministry of Health before March 2013, and China CDC, has developed and implemented an innovative program on TB control and prevention in China. Here we analyze data from a baseline survey in the three cities where the program was implemented: Zhengjiang City, Jiangsu Province; Yichang City, Hubei Province; and Hanzhong City, Shaanxi Province. They are located in the Eastern, Middle and Western Regions of China respectively, with a GDP per capital 73947 RMB, 56265 RMB, and 16935 RMB.

In 2012, the amount of registered TB patients in Zhengjiang, Hanzhong, and Yichang is 1768, 2336, and 3437, respectively, with an estimated non-compliance rate, caused by economic burden, 3.0 %, 3.3 % and 5.7 %. The primary service model is the integrated model. Tuberculosis suspects are provided a free diagnosis and anti-tuberculosis treatment, including designated first-line TB drugs (6 months for new patients, 8 months if previously treated), chest X-ray examination in the first and last months, and sputum smear test. The first-line TB drugs are isoniazid, rifampicin, rifampicin and butyl, pyrazinamide, ethambutol and streptomycin, and patients are requested to take tests and bring back their drugs every one or two month.

### Study sampling

Due to china’s large population and area, Chinese administration unit have consisted of several levels, mainly including provincial, prefectural, county, township, village. Prefectural level’s city, for instance Zhengjiang City, can also be divided into district, and further into street. To sample adapt to China’s administration unit, Probability-Proportional-to-Size Sampling (PPS) was adopted [39, 40]. Using selected key indicators, the required sample size per city was calculated to be 264 cases. This was rounded up to 270. As economic situation, to some extent, can stand for one place’s healthy development condition and people’s cognition of health. One county or district was randomly selected in each of three GDP per capita categories (high, middle and low). Six townships or streets were then sampled using PPS in each county or district and TB cases randomly sampled from the notification list. In total, 797 TB patients were surveyed (263 in Zhenjiang, 270 in Hanzhong and 264 in Yichang) and 60 of these were selected for in-depth interview.

### Data collection

Data collection employed a combination of both quantitative and qualitative methods. A survey questionnaire was developed which collected data on demographic and socio-economic information, patient diagnostic and treatment pathways and history, direct health service expenditures, indirect costs (transport and accommodation costs, associated additional household costs and household income foregone), borrowing resulting from illness, and compliance during treatment. We identified non-compliant patients as those who interrupted or stopped treatment (if not because of any adverse reactions) or refused treatment. The questionnaire and survey procedures were tested in a pilot study carried out in one project site. The survey was conducted by university students from Huazhong University of Science and Technology (Yichang), Xi’an Jiaotong University (Hanzhong) and Nanjing Medical University (Zhejiang). These students received appropriate training on interview skills and the contents of the questionnaire.

Quantitative data were self-reported and self-recalled. Household income was the aggregate of household income from production, wage incomes of household members, transfer income (pensions, remittances, welfare) and property income (interest, rent) [41–45]. Household income foregone was calculated by days unable to work since the onset of TB multiplied by the average reported lost income per day [29, 46]. The collection of direct and indirect costs followed the approach of Umar et al. [29].

Focus Group Discussions (FGDs) were held with TB patients. Each county organized one FGD with six to eight TB patients. A total of nine FGDs were conducted by university faculty members. All FGDs were tape recorded with the permission of participants. The inclusion criterion was simply defined in terms of patients with recent TB treatment experience who could clearly communicate their thoughts. As we identified patients from a list of TB patients compiled at the time that each patient began their TB regimen, there “recent” means six months or eight months as the standard course is six months for new cases and eight months for relapse cases. Each group was intended to reflect diversity in terms of gender, age-group and socio-economic status. Patients were selected with the help of the local CDC officials and informed about the study and its purpose. 60 of patients were selected, including 18 in Zhenjiang, 19 in Hanzhong and 23 in Yichang. The interviewer used a semi-structured interview guide with questions concerning direct health service expenditures, travel and subsistence costs, the affordability of TB services, and the reasons why treatment might be refused or interrupted.

### Major definitions

We define:

- OOP: out-of-pocket payment
- TOOP: total out-of-pocket payment, including direct medical costs, direct non-medical costs, and indirect costs, as a percent of household income;
- OOP2: out-of-pocket payment for diagnosis and treatment as a percent of household income;
- TLF: travel, lodging and food costs as a percent of household income;
- Irishr: income foregone due to the illness as a percent of household income;
- DSC: dietary supplement costs as a percent of household income;
- Ethel: patients’ subjective assessment whether TB causes a heavy burden to their family;
- Dietary supplement costs are expenditures on additional food, vitamins. During the period following diagnosis. Travel and subsistence costs are expenditures on travel, accommodation and food while seeking diagnosis and during treatment.

TOOP and OOP2 were defined as “catastrophic” if they equaled or exceeded 10 % [47, 48]. There is no generally accepted standard for excessive expenditure on TLF, IncFor and DSC. In this study, we defined TLF, IncFor and DSC as “heavy” for cases in which they equaled or exceeded their median values.

### Data management and analysis

Both the quantitative and qualitative data were entered into relational databases using the double-entry options of Epidata 3.1 and Nvivo 10, and analyzed with SPSS 20.0 and Nvivo10.

Following descriptive analysis, we used logistic regression to examine whether treatment compliance was associated with the extent of the economic burden. Taking account of the small sample of non-compliance patients, the bootstrap approach was adopted. Economic burden indicators are shown in Table 3. Additional variables included EcoBur and the household income level. Potential confounding variables are presented in Table 1. We first identified factors that might be expected to vary substantially between compliant and non-compliant patients and then included them in analysis using forward selection [49].

**Table 1:** Social-economic characteristics of TB patients

	Compliant (*n* = 765)	Non-compliant (*n* = 32)	*P* value*
Sex (female)	193(25.23)	10(31.25)	0.444
Age	56.07(15.02)	60.09(9.72)	0.031**
Residence			
Rural	704(92.03)	32(100)	0.225
Urban	59(7.71)	0(0)	
Rural- Urban continuum	2(0.26)	0(1)	
Education			
Primary school and lower	398(52.03)	27(84.38)	0.002
Secondary school or at the same level	265(34.64)	4(12.50)	
High school and higher	102(13.33)	0(0)	
Employment			
Employment	408(53.33)	15(46.88)	0.473
Unemployment	357(46.67)	17(53.12)	
Health insurance			
UEBMI	53(6.93)	0(0)	0.287
NCMS	679(88.76)	30(93.75)	
URBMI	21(2.75)	1(3.12)	
Household income			
Low (lowest 25)	184(24.31)	16(51.61)	0.003
Middle (medium 50)	372(49.14)	10(32.26)	
High (top 25)	201(26.55)	5(16.13)	
EcoBur			
Low	80(10.47)	0(0)	<0.001
General	289(37.83)	1(3.12)	
High	395(51.70)	31(96.88)	

Data are number of patients (%) or mean (SD). Several factors total number is not equal to 764 or 32, due to the existence of missing value. * This value calculated by chi-square test. **This value calculated by t-test

Almost everyone was covered by one of the health insurance schemes, with urban patients usually subscribing to UEBMI or URBMI and rural patients to NCMS. As indicated in Table 1, at the bivariate level we cannot reject the hypothesis that insurance type did not vary significantly between compliant and non-compliant patients. Because of its policy relevance we retain this variable in regression model. Finally, Age, UEBMI, URBMI and Education were therefore included in the regression model. As there were substantial correlations between all the economic burden variables, we fitted a separate model for each.

The data is self-reported and it is highly likely that there will be recall bias. To reduce the impact of data errors and exclude the influence of extreme values we transformed the continuous variables to generate dichotomous or ordinal forms. As this results in a substantial loss of information we also present findings using the original variables.

The qualitative data were analyzed using the ‘framework approach’ [50]. The framework was developed based on a topic guide, with categories emerging from an initial analysis of the transcripts and applied to the data to identify themes. All qualitative data were coded, sorted and classified in terms of this framework. Charting was used to identify common or divergent perceptions and explanations were developed. The Nvivo 10 software package was used to manage the data. All analysis was conducted in Chinese to avoid translation issues. The final results, including the major themes and verbatim quotes were then translated into English.

### Ethics statement

The Ethics Committee of China CDC gave final approval for this study (code: 201307). Written informed consent was obtained from all participants involved in the study. The ethics committee approved this procedure.

## Results

### Demographic, education, and household income characteristics of study subjects

Table 1 shows the patient characteristics including their demographic and economic status. There were almost three times as many males as females. Most patients lived in rural areas and participated in the NCMS. Around 52 % percent had only primary education and just under 47 % were unemployed. Almost 52 % considered TB as a heavy economic burden on their family. Only 32 patients reported poor compliance. Age, level of education and level of household income were significantly different between compliant and non-compliant patients.

### Medical expenditure and economic burden of TB patients

Table 2 shows the medical expenditures of TB patients. The average total expenditure on diagnosis and treatint for non-compliant patients is 17597 RMB (median 8400 RMB), and for compliant patients is 10146 RMB (median 5225 RMB). After reimbursed by insurance, the average OOP payment for diagnosis and treatment is 11164 RMB (median 4100 RMB) for non-compliant patients, and 7154 RMB (median 4000 RMB) for compliant patients. For non-compliant patients the average transportation cost and lodging and food costs is 247 RMB (median 164 RMB) and 881 RMB (median265 RMB), respectively, and for compliant patients the same costs is 251 RMB (median 114 RMB) and 526 RMB (median120 RMB), respectively. Compared with non-compliant patients, compliant patients’ average income reduction due to missed work is higher, with 385 RMB (median 0 RMB) VS 176 RMB (median 0 RMB), and the similar result for dietary supplement costs with 305 RMB (median 250 RMB) VS 289 RMB (median 200 RMB). Tested by nonparametric test, the foregoing results don’t have a significant difference between compliant and non-compliant TB patients.

**Table 2:** Direct and indirect costs of TB

	Compliant		Non-compliant		*P* value*
	Mean	Median [IQR]	Mean	Median [IQR]	
Total costs on diagnosis and treatment	10146	5225[2500–11638]	17597	8400[3000–30000]	0.084
OOP payment for diagnosis and treatment	7154	4000[2000–8000]	11164	4100[2500–11000]	0.356
Transportation cost (including companion members)	251	114[32–280]	247	164[39–365]	0.266
Lodging and food costs (including companion members)	526	120[0–640]	881	265[54–753]	0.248
Income reduction due to missed work days/hours	385	0[0]	176	0[0]	0.320
(including companion members)					
Dietary supplement costs	305	250[120–400]	289	200[200–500]	0.899
Sold	4083	2750[1500–8000]	6000	6000[6000–6000]	-
Borrow	10586	5000[3000–15000]	12515	4000[1500–20000]	-

Data are mean and median (IQR)

*This value calculated by nonparametric test

Table 3 shows the economic burden on TB patients. More than half had catastrophic health expenditure (TOOP ≥ 10 %). The economic burden caused by OOP2 was also heavy. Nearly half of patients faced a heavy economic burden due to TLF or DSC. Unexpectedly, the economic impact of income foregone was very limited compared with the results from other studies [17, 29]. It is possible that this concept was not well understood by respondents. The aforementioned burden generally had a higher proportion of those who were non-compliant, except for the burden caused by IncFor, and TOOP and TLF were significantly different between compliant and non-compliant patients. Using the unstratified continuous variables produced similar results (Table 4), except in the case of DSC, which became significantly different between compliant and non-compliant patients.

**Table 3:** Direct and indirect costs as % of household income of TB patients

	Compliant (*n* = 765)	Non-compliant (*n* = 32)	*P* value*
TOOP (*n* = 737)			
Heavy (=1)	479(67.56)	24(85.71)	0.043
OOP2 (*n* = 758)			
Heavy (=1)	427(58.41)	20(74.07)	0.104
TLF (*n* = 775)			
Heavy (=1)	368(49.20)	22(81.48)	0.001
IncFor (*n* = 789)			
Heavy (=1)	78(10.30)	2(6.25)	0.763
DSC (*n* = 521)			
Heavy (=1)	274(53.52)	7(77.28)	0.188

Data are number of patients (%). TOOP and OOP2 were defined as “catastrophic” or “heavy” if they equaled or exceeded 10 %, and TLF, IncFor and CDS were defined as “heavy” if it equaled to or exceeded its median.* This value calculated by chi-square test

**Table 4:** Direct and indirect costs as % of household income of TB patients*

	Compliant		Non-compliant		*p*-value**	*p*-value***
	Mean	Median [IQR]	Mean	Median [IQR]		
TOOP	71.74	20[7.67–50]	248.89	36.25[15.83–158.33]	0.001	0.006
OOP2	47.33	13.12[5.17–37.35]	164.43	20.23[7.91–93.66]	<0.001	0.058
TLF	8.38	1.63[0.32–6.52]	49.17	6.29[2–22.5]	0.001	0.003
IncFor	1.96	0[0–0]	0.44	0[0–0]	0.874	0.697
DSC	2.46	1[0.5–2.26]	4.67	4.29[2.04–6.94]	0.009	0.019
Household income	31336.52	22000[8000–41000]	19681.94	7860[2000–24500]	0.056	<0.001

*The variable is same with variable shown in Tables 3 and 5, but have not been transformed into categorical variable

**This value calculated by ANOVA based on log transformation

***This value calculated by ANCOVA based on log transformation, the covariate was Age, UEBMI, URBMI, and Education

The qualitative findings indicate that while basic TB drugs are provided free of charge, the costs of examinations, tests and adjuvant drugs can be substantial. There may also be overuse of CT scans and chest X-rays. This is illustrated in the following responses:

*“Within a month, I had two CT scans and a chest X-ray”*

*“…the cost is high, because of other checks, (such as) CT scans.”*

*“…drugs are free, but the examination fees will cost more than 230 (RMB) per month”*

*“…some drugs are free, but examination fees and other adjuvant drug fees are higher (than free drugs would have cost).”*

The qualitative results also show that, for longer courses of treatment, transport, lodging and food costs can be considerable:

*“Treatment time is long, (needing) eight or nine months… travel (cost) alone is high.”*

*“(Transportation, lodging and food costs) will probably be more than 1000 (RMB). Almost 10000 (RMB) a year.”*

*“…travel cost? Thousands of RMBs. (About) three or four thousands.”*

*“…like me, come here every month. (Every time I needed) at least 300 (RMB). Round-trip travel expenses will be at least 100 (RMB), (and this does) not include accommodation. … Nobody gives you reimbursement.”*

### The influence of a high economic burden on patient compliance

Logistic regression suggests that TB patient treatment compliance was related to TOOP, household income, EcoBur and TLF (Table 5). After adjustment for confounding factors the main findings were that: TB patients with middle level household incomes were less likely to occur non-compliance than those with low household income (OR 0.355, 95 % CI 0.140-0.830); those who felt EcoBur was general or high were more likely to occur non-compliance than those who felt it was low (OR 2.274, 95 % CI 1.081-10.928; OR 3.650, 95 % CI 1.278-12.346, respectively); and TB patients with high TLF were more likely to occur non-compliance than those with low (OR 4.150, 95 % CI 1.804-21.999). There was no significant difference in treatment compliance resulting from the economic burdens of OOP2, IncFor or DSC.

**Table 5:** Logistic regression of economic burden’s effect on TB patients’ compliance with OR and adjusted OR

	Pre-adjusted		Adjusted^a^	
	*p*-value	OR (95 % CI)	*p*-value	Adjusted OR (95 % CI)
TOOP (=1)	0.032	2.811(1.207–13.971)	0.07	2.406(0.939–12.244)
OOP2 (=1)	0.096	2.034(0.926–7.199)	0.18	1.822(0.766–6.013)
TLF (=1)	0.003	4.545(1.850–21.999)	0.001	4.150(1.804–21.999)
IncFor (=1)	0.396	0.580(0.000–1.697)	0.653	0.829(0.000–2.754)
DSC (=1)	0.094	3.040(0.689–20.589)	0.155	2.622(0.500–25.815)
Household income				
Low (lowest 25 %)				
Middle (medium 50 %)	0.001	0.309(0.121–0.657)	0.011	0.355(0.140–0.830)
High (top 25 %)	0.008	0.286(0.061–0.709)	0.155	0.419(0.066–1.435)
EcoBur				
Low				
General	0.047	2.300(1.091–8.987)	0.037	2.274(1.081–10.928)
High	0.002	3.333(1.113–10.989)	0.001	3.650(1.278–12.346)

^a^Adjusted for the variable Age, UEBMI, URBMI, and Education

Similar results were obtained with the untransformed continuous variables allowing for the effects of age, UEBMI, URBMI, and education, as shown in Table 4. Again, the effect of expenditure on DSC became significant.

Qualitative findings suggest that, for some patients, even the burden of transport costs can influence their treatment compliance, especially for families with more than one TB patient.

*“… (The) travel cost is high, (and) influenced me (in terms of compliance)”*

*“There is a family, all (have) tuberculosis. If they were examined, in fact, they could be cured, but (because they) don’t (have) money they are unwilling to travel (for treatment).”*

## Discussion

In this study, we found that age, education level, household income and patients’ subjective assessment whether TB causes a heavy burden to their family are significant different between group compliance and non-compliance. In consistence with previous studies, we also got the similar result that patients who are elderly, low education level are more likely to non-compliance to TB treatment [8, 9, 14, 15]. The elderly and low education level patients are more likely to have a limited ability to get and understand related TB knowledge. This cue that except the chemotherapy, a humanist intervention or assistance may be needed.

Compared with direct medical costs, the non-medical costs, dietary supplement costs and indirect costs were not high. This finding is not consistent with other studies, which often indicate that the main burden arises from transport, diet supplements and loss of income [17, 46]. Explanation is that most of the patients were located in rural areas with incomes mainly coming from farming or self-employment (Table 2). The time required for diagnosis and treatment have little impact on the time available for work and the food for diet supplementation usually be home grown.

Though the free-TB service policy, patients have to pay a high expenditure on diagnosis and treatment. Even though covered by insurance, this expenditure doesn’t reduce too much. In the integrated model, TB patients can only be diagnosed and treated under the free treatment policy in the designated hospital. The free treatment policy just covered first-line anti-TB drugs, TB sputum smears and cultures, and X-ray examinations for the first and last months [16, 21, 23, 24, 51], and these items’ profit is low. Driven by the revenue, doctors may over-prescript additional drugs and test [16, 33]. As the qualitative results showed, overuse of liver protection drugs and CT test is not uncommon. And also previous studies revealed that providers have the motivation to use second-line anti-TB drugs [16, 21, 26]. All these overused items neither covered by the free policy, nor by the insurance, resulting in high patient OOP medical expenditure. A systematic review based on 85 research articles found no reliable evidence to support prescription of drugs protects the liver [52]. studies also reported that NCMS did not reduce patient out-of-pocket costs per episode [31, 53, 54]. All of these indicated that improving providers’ salary system, expanding insurance’s coverage is needed.

This study revealed a very high economic burden for TB patients in China, in line with prior studies [16, 28, 29, 55]. Almost 68 % of TB patients reported catastrophic health expenditure (Table 3), much higher than the average for China [43, 44]. Patients with low household incomes or who felt that the economic burden was high were more likely to occur non-compliance (Table 5). These findings lend support to the argument that there is an association between TB and poverty and suggests that TB patients still face economic barriers in seeking diagnosis and treatment [20, 28, 30].

Contrary to expectations, the quantitative analysis suggests that a high economic burden did not in itself result in poor compliance (Table 5). This finding has two possible explanations. First, a sunk cost effect probably exists in relation to seeking diagnosis and treatment. If a patient has spent a lot of money at early stage, he or she may continue to seek treatment, to avoid that expenditure becoming a sunk cost, even borrowing money or selling property to continue treatment. Second, in the process of calculating the economic burden, we only include the actual costs, not the anticipated costs. Patients with low household incomes may interrupt their treatment because they expect that further treatment will become unaffordable [9]. Such patients would certainly feel that the economic burden of treatment was very high. And these reasons can also explain why we can’t get a significant result from the test of direct and indirect costs between compliant patients and non-compliant patients.

Even the costs related to transport, accommodation and food could lead to catastrophic expenditure (Table 3). And the results from both from quantitative and qualitative studies demonstrate that the burden from costs on transport, lodging and food had a substantial effect on treatment compliance, in line with Tadesse’s study [3].This also lend supports to the hypothesis above. In China, under the free policy, providers are supposed to educate TB patients related knowledge before chemotherapy. Previous studies indicated several points: lack of knowledge about anti-TB treatment was associated with higher non-compliance rates [8, 13]; some patients still confused about treatment regimen after health education [8]; adverse reactions contribute to non-compliance [15, 21]. For patients with low household income, if adverse reactions occurred or symptoms relieved and can’t convenient keep in touch with doctors, travelling long distances to a health care facility at considerable expense will challenge their willingness to continue treatment [3, 16]. Another situation is that as patients have to bring back their medicine monthly, the high cost of transport, lodging and food really impede them from doing so.

What can we do to improve TB patients’ compliance? We suggest we can through two aspects to influence patients’ behavior: knowledge and economic burden. As we discussed before, for patients, transport, accommodation and food costs can be a barrier to keep in touch with doctors. So one of the useful ways is to develop mobile phone based intervention, as several studies already emphasize this intervention’s potential effectiveness [56–58]. Another way is to expand the current package to cover patients’ transport, accommodation and food costs or offer related economic assistance. Previous studies indicated that lack of knowledge about anti-TB treatment was associated with higher non-compliance rates [8, 13], and some patients still confused about treatment regimen after health education [8]; we found that patients who are elderly, low education level are more likely to non-compliance to TB treatment. This implied that a humanist intervention tailored by these factors may be helpful.

For economic burden, no matter actually happened burden or anticipated burden, an experience-based opinion is that due to the free-TB service policy, the economic burden caused by OOP2 just happened when patients already played a visit to doctor, otherwise they wouldn’t know or anticipate whether the burden is high. According to the above discussion, improving doctors’ salary system to cut off the revenue-oriented incentive, and expanding current insurance’s coverage is useful. Chinese insurance is a multilevel system. In our study, the actually happened economic burden is high. To enhance the cooperation between different level’s insurance, for instance between basic insurance and critical illness insurance or assistance, is necessary.

### Limitations

The proportion of non-adherence in our study is not high compared with that were reported in previous studies [8–11], and much of the difference may be due to differences in study design and definition of non-adherence to anti-TB treatment. As we want to evaluate economic burden’s effect, we identified non-compliant patients as those who interrupted or stopped treatment (if not because of any adverse reactions) or refused treatment. We identified patients from a list of TB patients compiled at the time that each patient began their TB regimen. Some of patients may not be on this list, especially those located in remote rural areas, who are more likely to be non-compliant because of the long distance to the health care facility. In addition, suspected TB patients were not included in our study. So, as a matter of fact, the situation is more serious, and the effect of economic burden on compliance may therefore be underestimated.

In our study, we included three sample cities, located in the Eastern, Middle and Western regions of China, respectively. Background of the studied setting is different more or less, and the more appropriate analysis is disaggregate patients by the three regions. Due to the limitation of sample size of non-compliance patients, this method will come with high risk of deviation. Further study with more sample size is needed to explore inter-regional difference.

To avoid the influence of inaccurate data and extreme values and to simplify interpretation of the regression results, we transformed several continuous variable to ordered category form, though this had little impact on the results. However, recall bias will still almost certainly have influenced the findings. The economic impact of income foregone was very limited compared with the results from other studies [17, 29]. It is possible that this concept was not well understood by respondents.

## Conclusion

Our results lend weight to the argument that there is a link between TB and poverty and that TB patients continue to face economic barriers in seeking diagnosis and treatment. TB patients with low household incomes or who feel that the economic burden of treatment is high are more likely to have poor compliance. As indicated above, the number of TB patients in China is very large. The provision of free anti-TB drugs has been a valuable step forward but to improve the coverage and effectiveness of control programs for TB. We suggest that the useful way is to develop mobile phone based intervention or offer patients transport, accommodation and food economic assistance to break down the barrier of keeping in touch with doctors. Another way is to improve doctors’ salary system to cut off the revenue-oriented incentive, expand current insurance’s coverage, and enhance cooperation between different level’s insurance.

## Additional file

Additional file 1:
**Multilingual abstracts in the six official working languages of the United Nations.** (DOC 21 kb)

## Abbreviations

CDC: Centre for Disease Control
DOT: Direct observation of treatment
DSC: Dietary supplement costs as a percent of household income
EcoBur: Patients’ subjective assessment whether TB causes a heavy burden to their family
FGDs: Focus group discussions
GDP: Gross domestic product
IncFor: Income foregone due to the illness as a percent of household income
NCMS: New Cooperative Medical Scheme
NHPFC: National Health and Family Planning Commission of the People’s Republic of China
OOP: Out-of-pocket payment
OOP2: Out-of-pocket payment for diagnosis and treatment as a percent of household income
PPS: Probability-Proportional-to-Size Sampling
TB: Tuberculosis
TLF: Travel, lodging and food costs as a percent of household income
TOOP: Total out-of-pocket payment, including direct medical costs, direct non-medical costs, and indirect costs, as a percent of household income
UEBMI: Urban Employee Basic Medical Insurance
URBMI: Urban Resident Basic Medical Insurance
WHO: World Health Organization
