# Supplementary material for: Are free anti-tuberculosis drugs enough? An empirical study from three cities in China
Source: Infect Dis Poverty. 2015 Oct 28;4:47. doi: 10.1186/s40249-015-0080-y (PMC4625923; doi:10.1186/s40249-015-0080-y)
Supplement: Additional file 1: — Multilingual abstracts in the six official working languages of the United Nations. (DOC 21 kb) [file 40249_2015_80_MOESM1_ESM.doc]

**免费抗结核药物足够吗？一个来自中国三市的实证研究**

**陈山泉^1^，张慧^2,8*^，潘瑶^3^，龙倩^4,5^，项莉^6^，姚岚^6^，Henry Lucas^7^**

**1 赛马会公共卫生及基层医疗学院，香港中文大学，香港，中国**

**2 结核病预防控制中心，中国疾病预防控制中心，北京，中国**

**3 中山大学附属第三医院，广州，中国**

**4 杜克全球卫生研究所，杜克大学，****达勒姆市，北卡罗来纳州，美国**

**5 全球健康研究中心，杜克昆山大学，昆山，中国**

**6 医药卫生管理学院，华中科技大学，武汉，中国**

**7发展研究所，****苏塞克斯大学，布莱顿市，英国**

**8 中国疾病预防控制中心，北京，中国**

**背景：**在中国，结核病患者在寻求诊断和治疗的过程中仍然面临着许多困难。有证据表明，结核病患者及其家庭的沉重疾病经济负担对治疗的依从性将产生不利的影响。

**方法：**本研究在中国三个城市开展了横断面调查。运用PPS抽样，基于农村的乡镇或城市的街道，整群抽取结核病患者。通过问卷调查的方式搜集患者数据，并运用结核病患者的关键人物访谈和焦点小组访谈，来进一步了解结核病患者的疾病经济负担，及由此疾病经济负担给其治疗依从性带来的影响。

**结果：**本研究总共调查了797个结核病患者，其中60名结核病患者进行了深度访谈。超过一半的结核病患者发生了灾难性卫生支出。家庭收入较高的结核病患者发生非依从的情况较少（OR 0.355, 95 % CI 0.140–0.830）。主观评价认为结核病诊治所导致的疾病经济负担较为沉重的结核病患者治疗依从性较差（OR 3.650, 95 % CI 1.278–12.346）。直接非医疗费用（交通费、食宿费）较高的结核病患者治疗依从性较差（OR 4.150, 95 % CI 1.804–21.999）。定性调查的结论与问卷所获得的定量数据得出的结论一致。

**结论：**在中国，因寻求诊断和治疗而导致的疾病经济负担依旧是结核病患者的一个难题。通过减少结核病的诊治费用、给予交通和食宿费用的补助等方式可以提高结核病患者的治疗依从性。完善结核病医护人员的薪酬机制，从而减少其趋利行为；扩展目前医疗保障制度的覆盖面等，均可以有效的减轻患者实际的疾病经济负担及其期望的疾病经济负担。

**关键词**：结核病，经济负担，灾难性卫生支出，依从性，中国
